# A Noninvasive Fecal-DNA Detection Method for Sarcoptic Mange in the San Joaquin Kit Fox (*Vulpes macrotis mutica*)

**DOI:** 10.3390/ani16172740

**Published:** 2026-09-02

**Authors:** Anaisía N. Brown, Stevi L. Vanderzwan, Erica C. Kelly, Jaime L. Rudd, Deana L. Clifford, Brian L. Cypher, Benjamin N. Sacks

**Affiliations:** 1 Mammalian Ecology and Conservation Unit (MECU), Veterinary Genetics Laboratory, School of Veterinary Medicine, University of California—Davis, One Shields Avenue, Davis, CA 95616, USA; ab4575@gmail.com (A.N.B.); svanderzwan@ucdavis.edu (S.L.V.); 2 Endangered Species Recovery Program, California State University-Stanislaus, 1 University Circle, Turlock, CA 95382, USAjrudd@esrp-ca.org (J.L.R.); bcypher@esrp.org (B.L.C.); 3 Wildlife Health Laboratory, California Department of Fish and Wildlife, 1701 Nimbus Rd, Rancho Cordova, CA 95670, USA; deana.clifford@wildlife.ca.gov; 4 Department of Population Health and Reproduction, School of Veterinary Medicine, University of California—Davis, One Shields Avenue, Davis, CA 95616, USA

**Keywords:** California, DNA metabarcoding, endangered species, fecal DNA, kit fox, mange, *Sarcoptes scabiei*, surveillance, *Vulpes macrotis mutica*

## Abstract

Endangered San Joaquin kit foxes (*Vulpes macrotis mutica*) living in Bakersfield and Taft, California, have declined due to an ongoing sarcoptic mange epizootic. Population monitoring for signs of sarcoptic mange typically involves camera surveillance in these urban populations, but in unmonitored non-urban populations, where camera monitoring is less practical, sarcoptic mange may go undetected. A noninvasive fecal DNA assay would be beneficial in such areas. Because mange mites induce pruritus (itching), they may elicit grooming and ingestion by foxes, enabling the development of a noninvasive fecal-DNA detection method targeting sarcoptic mite DNA in kit fox scats. To evaluate the utility of a fecal-DNA detection method, we tested quantitative (q) PCR and DNA metabarcoding methods aimed at detecting mange mite DNA in fecal samples collected from confirmed and putative infested San Joaquin kit foxes and non-infested foxes. Both methods demonstrated high specificity, estimated at 100% (95% CI: 92–100%), but low sensitivity among all cases (confirmed, putative), specifically 11% (95% CI: 0.04–0.24) for qPCR and 22% (95% CI: 0.11–0.37) for metabarcoding. However, nine of the 11 (82%) confirmed cases tested positive, possibly because most of the putative cases had lower mite burdens or were misdiagnosed. Fecal metabarcoding could represent a useful adjunct to camera traps for sarcoptic mange monitoring in kit fox populations and, pending additional trials, potentially other wildlife as well.

## Simple Summary

Endangered San Joaquin kit foxes (*Vulpes macrotis mutica*) have declined in some urban locations due to an ongoing sarcoptic mange epizootic, raising concerns that the disease could spread to other populations. Monitoring urban populations involves placement and regular checking of cameras and live trapping to detect and treat mange cases, but these methods are less feasible in some non-urban populations. Therefore, we sought to develop a noninvasive method of detecting mange in kit fox scats collected from the environment. Our approach depends on kit foxes ingesting the causative *Sarcoptes scabiei* mite during grooming, which is detected through DNA passed through the digestive system. We tested PCR-based approaches to detecting sarcoptic mite DNA in kit fox scats collected from confirmed infested, putative infested (based on visual inspection), and non-infested San Joaquin kit foxes. We obtained no false-positive results, but only detected mite DNA in 22% of the scats from total confirmed plus putative mange-infested foxes. However, nine of the 11 (82%) confirmed infested foxes tested positive. Possibly, most of the putative cases had lower mite burdens or, in some cases, may have been misdiagnosed. Fecal metabarcoding could represent a cost-effective alternative to surveying for mange on a population level.

## 1. Introduction

Sarcoptic mange, caused by the sarcoptic mite (*Sarcoptes scabiei*), can be a significant parasite of canids and other mammalian hosts; it presents as dermatitis, pruritus (itching), erythema, lichenification (skin-thickening), and sometimes black skin discoloration [1,2]. Since its emergence in Bakersfield, California, USA in 2013, a sarcoptic mange epizootic has proven especially detrimental to the San Joaquin kit fox (*Vulpes macrotis mutica*) [3,4].

San Joaquin kit foxes are a federally endangered species endemic to the San Joaquin Valley in California [5]. Within the San Joaquin Valley, there are three core kit fox populations, one large urban population, and fewer than a dozen smaller satellite populations [3,6]. Fewer than 5000 individuals remain in all. Bakersfield, California, a highly urbanized area, has one of the largest populations and the highest density of urban San Joaquin kit foxes [6,7]. However, a sarcoptic mange epizootic led to a decline of nearly 67% in Bakersfield’s San Joaquin kit fox population in the decade beginning in 2013 [3,6]. In 2019, the disease was also identified in the neighboring town of Taft.

Without human intervention, sarcoptic mange is thought to result in a 100% mortality rate among San Joaquin kit foxes [3]. Depending on the severity of the disease, treatment involves either live trapping infested kit foxes and applying acaracide medication directly in the field or, for more advanced-stage mange cases, foxes are sent to a rehabilitation facility for intensive treatment and supportive care. Even after treatment, kit foxes, like other vulpids, experience reinfestation because they lack immunity to the disease [3,8]. Thus, intensive surveillance for sarcoptic mange is needed to detect emergence in a population, followed by aggressive intervention efforts to prevent establishment.

Current methods to detect sarcoptic mange rely on camera surveillance and live trapping to collect skin scrapings. The skin scrapings are examined microscopically for the presence of sarcoptic mites [3,6]. Live trapping, which is only used after a report of a suspected case of mange is substantiated by camera surveillance or direct observation, is time- and labor-intensive and can also induce stress in the animals. We sought to develop and evaluate a noninvasive detection method for sarcoptic mange that could be paired with or serve as an alternative to current detection methods for unmonitored, non-urban populations of kit foxes. These populations are not easily observed, and the presence of mange remains unknown as infested foxes may go unreported.

Mange infestation, which may induce excessive grooming behaviors in carnivores, including kit foxes [7], can be detected in feces of infested individuals presumably due to ingestion of mites and excretion of mite DNA [9,10]. Consequently, noninvasive tests can be used to detect mange mite DNA in carnivore feces [9,10,11]. To be effective, assays must be highly specific and sufficiently sensitive to detect mite DNA in at least some infested individuals from a population sample. Previous studies have found near perfect specificity but lower sensitivity both for notoedric mange in bobcats (*Lynx rufus*) at 52.6% (95% CI: 29–76%) [9] and sarcoptic mange in gray wolves (*Canis lupus*) at 30% (95% CI: 14–53%) [10]. However, the application of such tests on the population level can be extremely useful for detection of mange because the probability of obtaining a positive test increases predictably as a function of sensitivity, specificity, and prevalence in the population [12,13]. For example, a test with 30% sensitivity (and 100% specificity) tested on 100 scats from a population with 10% prevalence would imply a detection probability of 97%.

In this study, we quantified specificity and sensitivity as applied to kit fox scats of the quantitative (q) PCR assay used previously for wolf scats [10] and compared these metrics to those based on the use of an alternative method involving sequencing, known as metabarcoding. Both assays used primers intended to be species-specific that target the 16S mitochondrial gene in *S. scabiei* [11]. In contrast to PCR followed by gel electrophoresis or qPCR, metabarcoding results in complete sequencing of amplicons representing individual molecules, effectively eliminating the possibility of false-positives due to non-specific amplification. We tested both methods on serial dilutions of sarcoptic mite DNA and fecal samples from known (or putative) infested and uninfested foxes. Thus, while our primary objective was to assess the utility of a fecal-DNA-based test for mange in general, the secondary objective was to determine which approach performed better (qPCR vs. metabarcoding).

## 2. Materials and Methods

### 2.1. Samples and Experimental Design

We tested qPCR and DNA metabarcoding assays for sarcoptic mite DNA using DNA of known concentrations as positive controls, negative controls, and fecal DNA samples extracted from scats of foxes determined based on physical examination assessing if they were infested or uninfested with mange (known-status samples).

The positive controls used throughout the study were DNA from a sarcoptic mite collected from a skin scraping of a mange-infested San Joaquin kit fox from Bakersfield. To determine the detection threshold for sarcoptic mite DNA, we quantified the DNA in the extract (28.6 ng/µL) using a Qubit fluorometer (Qiagen, Inc., Germantown, MD, USA) and prepared serial dilutions corresponding to concentrations ranging 0.001–5.0 ng/µL. Negative controls contained no sarcoptic mite or fecal DNA, but included all other reagents, along with TE Buffer (10 mM Tris-HCl + 1 mM EDTA) to equilibrate reaction volume to that of positive controls and fecal DNA samples. For the qPCR, we established a standard curve using the positive controls, which indicated exponential amplification between 27 and 37 PCR cycles.

We included in each qPCR and metabarcoding batch of samples (*n* = 96, including controls) a subset of these positive controls as well. For qPCR, we used positive controls at 0.625 ng/µL, 0.039 ng/µL, 0.0195 ng/µL, 0.005 ng/µL, and 0.001 ng/µL, along with one negative control. Based on preliminary qPCR tests, we dropped the lowest-concentration positive control (0.001 ng/µL) from our metabarcoding protocol and included only the 4 higher concentrations (0.625 ng/µL, 0.039 ng/µL, 0.0195 ng/µL, 0.005 ng/µL) along with 4 negative controls for each batch.

Fecal samples were collected directly from 65 San Joaquin kit foxes confirmed or suspected of being infested (*n* = 45) and uninfested (*n* = 20) with mange (Table S1). Putative infested foxes (*n* = 34) were diagnosed based on coat appearance and the presence of mange-like lesions (e.g., bilateral alopecia of the ischial tuberosities, extremities, and tail with flaking, erythema, and thickened/crusted skin), whereas confirmed infestations were diagnosed based on the presence of mange-like lesions and positive skin scrapings (*n* = 11) in which *S. scabiei* mites were observed microscopically. Foxes that appeared clinically healthy and lacking mange-like lesions were considered non-infested (*n* = 20). Although we did not collect data on mite burden, a mange scoring system was used to document the severity of mange-like lesions [14]. Most cases suspected based on visual inspection were mild (Class I); therefore, they potentially had lower mite burdens than the confirmed cases, which had more advanced signs (Class II or III). Most scats were preserved in a −20 °C freezer for up to 12 years (Table S1). Additionally, we supplemented our uninfested sample with scats from San Joaquin kit foxes located in the Ciervo–Panoche population (*n* = 24), the most geographically distant (51 km) population from Bakersfield and Taft, a region where mange has not been documented and was presumed absent (Table S1). Our assumption was conservative with respect to estimation of specificity in that an infested fox that tested positive would have been recorded in our estimates as a false-positive.

The qPCR and metabarcoding runs (batches of 96 samples and controls) were separated by population (i.e., Ciervo–Panoche vs. Bakersfield) and by known infested versus uninfested samples to reduce potential for cross-contamination. Samples and controls were run in triplicate. Samples were considered positive if ≥1 replicates tested positive.

### 2.2. Fecal DNA Extraction

Laboratory analyses were conducted at the Mammalian Ecology and Conservation Unit (MECU) of the Veterinary Genetics Laboratory at the University of California, Davis. We extracted DNA from 200 mg of fecal sample using the Stool DNA Purification kit (EURx, Gdansk, Poland). This kit also contained reagents to remove or neutralize PCR inhibitors. Extraction procedures followed the manufacturer’s protocol.

### 2.3. qPCR

We adopted a modified version of the qPCR protocol used by Angelone-Alasaad et al. [11] to identify sarcoptic mite DNA. We used the same primers SSUD-F (5′-GGGTCTTTTTGTCTTGGAATAAA-3′), SSUD-R (5′-CTAAGGTAGCGAAATCATTAGC-3′) and TaqMan Universal PCR Master Mix (Applied Biosystems, Foster City, CA, USA), and the same TaqMan probe (5′-GGTAACTTGTATGAAGGGACTAACTAAA-3′) with 5′ Dye 6FAM reporter, except that we used a QSY Probe 3′ quencher instead of the BHQ1 quencher (Applied Biosystems, Foster City, CA, USA).

Our qPCR reaction consisted of 0.8 μM of SSUD-F and SSUD-R primers, 0.25 μM of custom TaqMan Probe, 1× of TaqMan Universal PCR Master Mix (Applied Biosystems, Foster City, CA, USA), and 1.5 μL of DNA of varying or unknown concentrations (or TE buffer instead for NTCs). We used a QuantStudio 3 Real-Time qPCR System (Applied Biosystems, Foster City, CA, USA) with a thermocycling profile as follows: denaturation at 95 °C for 10 min and amplification for 45 cycles at 95 °C for 15 s and at 60 °C for 1 min.

### 2.4. DNA Metabarcoding

The metabarcoding procedure included two rounds of PCR, one for amplifying target mite DNA (amplicon PCR) and the other for adding unique indexes to each sample before pooling for sequencing (index PCR). Each batch of samples included four NTCs and four positive controls (0.625 ng/µL, 0.039 ng/µL, 0.0195 ng/µL, 0.0050 ng/µL). We developed DNA metabarcoding primers based on the same PCR primers indicated above (P5_SSUDF, P5_SSUDR) targeting the 16S region of the mitochondrial genome of sarcoptic mites [11]. Additionally, however, we included 5′ overhangs on P5_SSUDF (5′ TCTTTCCCTACACGACGCTCTTCCGATCTGGGTCTTTTTGTCTTGGAATAAA-‘3) and P7_SSUDR (5′-GTGACTGGAGTTCAGACGTGTGCTCTTCCGATCTCTAAGGTAGCGAAATCATTAGC-3’) to allow for annealing of indexed Illumina-compatible primers during the index PCR step.

Amplicon PCR consisted of 0.2 µM of P5_SSUDF and P5_SSUDR primers, 2.18 µL of RNase-free water, 2 µL of DNA (TE buffer for NTC), 1× Qiagen Multiplex PCR Mastermix, and 1× Qiagen Q-solution (Qiagen, Valencia, CA, USA) in an 11 µL reaction. We used 2720 thermal cyclers (Applied Biosystems, Foster City, CA, USA) with a thermal cycling profile of 95 °C for 15 min, followed by 45 cycles of 94 °C for 30 s, 53 °C for 45 s, 72 °C for 1.5 min, and a final elongation step at 72 °C for 7 min.

Following amplicon PCR was the Exo-SAP purification step. The overall reaction volume was 6 µL with 3 µL of amplicon PCR product, 2.78 µL of 1× TE buffer (10 mM of Tris-HCl + 1 mM of EDTA), 1.5 units of Exonuclease I, and 0.15 units of shrimp alkaline phosphatase (rSAP). The thermocycling profile for Exo-SAP was 37 °C for 30 min, followed by 80 °C for 15 min, completed in one cycle.

The next step was index PCR, which used 5 µL of RNase-free water, 1× NEBNext Ultra II Master Mix (New England Biolabs, Ipswich, MA, USA), 1 µM of Custom i5 primer, 1 µM of P7 index primer, and 3 µL of Exo-SAP-purified PCR product in a 25 µL reaction. We used 2720 thermal cyclers (Applied Biosystems, Foster City, CA, USA) and the thermal profile was 98 °C for 30 s, 98 °C for 10 s, 53 °C for 45 s, and 72 °C for 1.5 min, after which the following steps would be repeated for eight cycles, then 65 °C for 5 min.

We pooled 2 µL of each index-PCR product and cleaned the pool with the QiaQuick PCR Purification kit following the manufacturer’s protocol (Qiagen, Valencia, CA, USA). The purified pooled DNA concentrations were quantified using a Qubit fluorometer (Qiagen, Inc., Germantown, MD, USA) and sent for sequencing on an Illumina Novaseq X Plus 10B lane (150 bp paired-end reads) at Admera Health Services, Inc. (South Plainfield, NJ, USA).

### 2.5. Bioinformatics for Metabarcoding Data

We used Cutadapt to trim adapter sequence and remove low-quality reads from the metabarcoding sequences [15]. We pulled out and quantified (i.e., counted) the amplified sequence variants attributable to each sample or control using DADA2 [16]. We determined which sequence corresponded to the target mange mite using the Basic Local Alignment Search Tool (BLAST; v 2.17) in GenBank [17]. We confirmed that the sequence matching the *S. scabiei* reference sequence in GenBank (100 percent sequence identity and overlap) was the one that composed most of the sequencing reads. We discarded all other sequences, most of which were trace amplification of bacteria or other fecal contaminants.

Positive test results were assessed for samples in both qPCR and metabarcoding according to quantitative cutoffs determined based on the negative and positive controls. For qPCR, we used a maximum cycle threshold (CT) consistent with performance of positive controls and not negative controls, and in metabarcoding, we used a count of *S. scabiei* reads above which all positive controls and no negative controls exceeded. Specifically, we used as the metabarcoding threshold 2× the highest number of *S. scabiei* sequencing reads observed in a negative control sample. A small number of sequencing reads was expected in some negative controls due to trace amounts of cross-contamination or barcode hopping during PCR or sequencing, respectively [18].

### 2.6. Data Analysis

We estimated sensitivity (Se), the probability that a mange-infested individual tests positive, and specificity (Sp), the probability that a non-infested individual tests negative, using the R programming software (v 4.4.1) package epiR [19]. The epiR statistical package provided sensitivity and specificity point estimates and exact binomial confidence intervals.

We then used the estimates of sensitivity and specificity to infer an unbiased estimate of true prevalence in a population from the cumulative set of tests conducted. Specifically, we calculated the unbiased (i.e., true) prevalence based on the apparent prevalence (AP) and the estimated sensitivity and specificity of the tests using the following calculation [12,13]:

unbiased (true) prevalence = (AP + Sp − 1)/(Se + Sp − 1).(1)

We used this relationship also to estimate the probabilities of detection in a population as a function of sample size (no. of scats tested) and prevalence (i.e., true prevalence) given the estimated Se (conservatively assuming no false-positives) as follows:

detection probability = 1 − (1 − Se)^n^^*TP^, (2)

where *n* = number of scats tested and TP = the true prevalence.

## 3. Results

### 3.1. qPCR

Positive controls with concentrations ≥ 0.039 ng/µL amplified successfully in all three replicates, while negative controls and positive controls with concentrations ranging 0.001–0.0195 ng/µL failed consistently. For CT counts ranging 27 to 37, the logarithm of the concentration (ng/µL) was strongly inversely correlated with CT count (*r* = −0.99) and could be estimated based on the following regression formula: ln(conc) = 16.66 − 0.529 × CT.

The qPCR results indicated that five of 45 samples from putative and confirmed infested kit foxes and all 44 samples from uninfested individuals accurately matched the presumed infestation status of the kit foxes (Table S2). Correspondingly, the sensitivity of the qPCR assay was estimated at 11% (95% CI: 0.04–0.24), and its specificity was estimated at 100% (95% CI: 0.92–1.00). Of these five positive-testing samples, four of them tested positive at all three replicates and one tested positive for only one replicate. Considering only the 11 foxes confirmed by skin scraping to have active cases of mange, sensitivity was much higher at 45.5% (95% CI: 0.17–0.77), with all five positive tests corresponding to one of these confirmed cases. None of the putative cases tested positive.

Based on the conservative estimate utilizing all 45 putative or confirmed mange-infested foxes, the true prevalence values are expected to be nearly 10-times higher than what would be estimated based on the qPCR testing of scats. These relationships imply a detection probability in a population with 10% prevalence of 69% for 100 scats or 90% for 200 scats.

### 3.2. Metabarcoding

**Figure 1:**
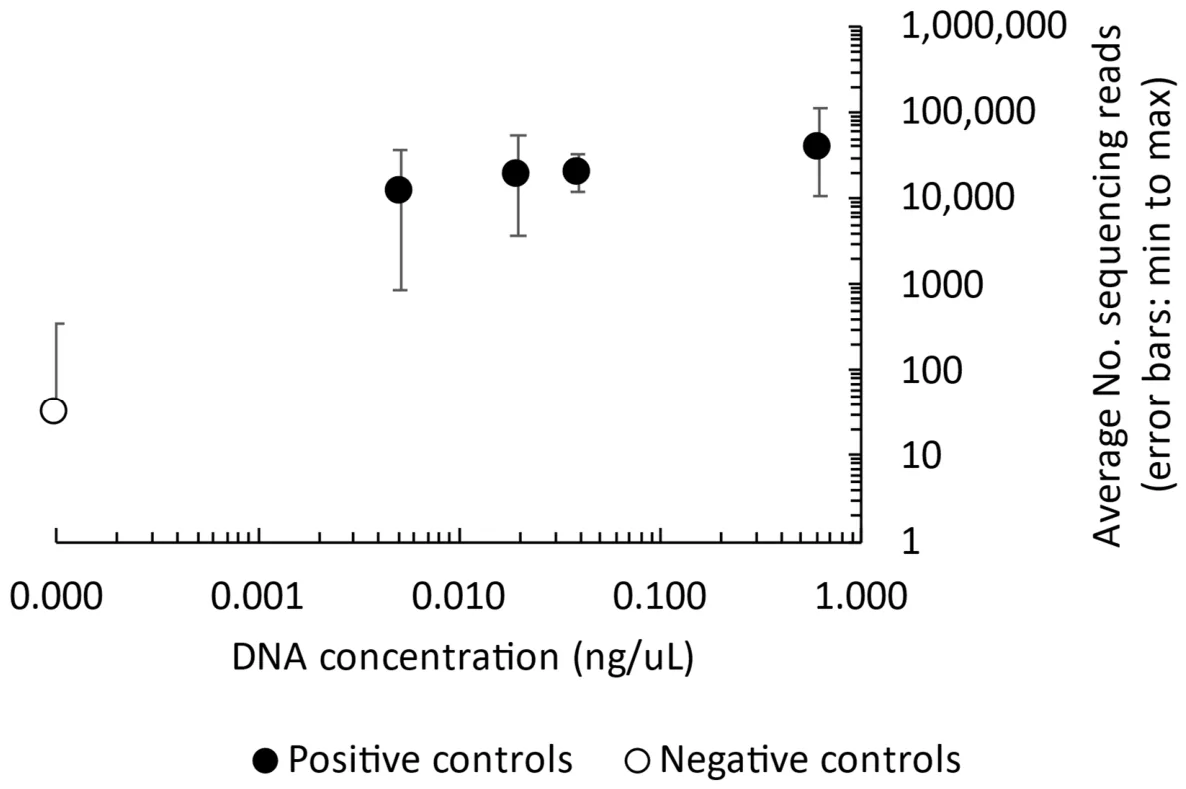
The number of *Sarcoptes scabiei* reads detected in the negative and positive control samples of the metabarcoding assay. The highest number of *S. scabiei* sequencing reads reflecting trace background contamination in a negative control (*n* = 20) was 357; the threshold value was therefore set at 714 reads. The fewest number of reads detected at the 0.005 ng/µL-concentration positive control (*n* = 4) was 858.

The number of *S. scabiei* reads in negative controls averaged 30.8 reads each and ranged from zero to 357 (Table S3). Therefore, the threshold to exceed for a positive test was set to 714 sequencing reads. The number of reads observed in positive controls ranged from 858 to 113,256 (Figure 1). Thus, we detected sarcoptic mite DNA in concentrations as low as 0.005 ng/µL, which was lower than what we detected in the qPCR test. For all positive controls (i.e., concentrations ≥ 0.005 ng/µL), all three replicates tested positive.

**Figure 2:**
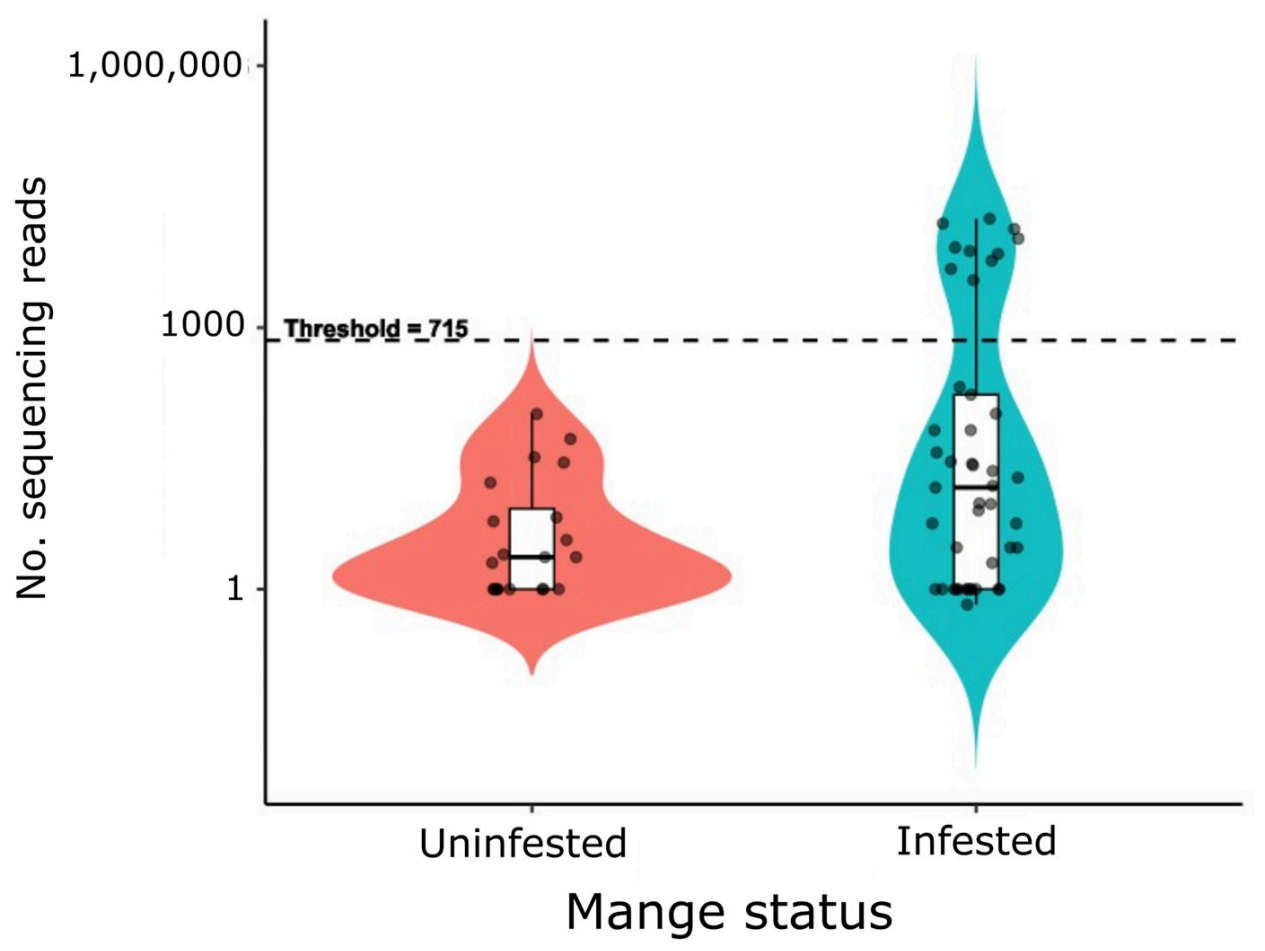
Numbers of *Sarcoptes scabiei* sequencing reads detected with metabarcoding for individuals that were uninfested (*n* = 44) and known or presumed to be infested (*n* = 45) with mange. The threshold for an individual identified as positive was ≥715 reads.

All samples from known uninfested foxes (*n* = 20) and presumed uninfested kit foxes from the Ciervo–Panoche population (*n* = 24) had fewer than 714 *S. scabiei* reads and therefore tested negative, indicating a specificity estimate of 100% (95% CI: 0.92–1.00) in the sample (Figure 2). Of the samples from putative or confirmed infested kit foxes (*n* = 45), 10 tested positive, corresponding to a sensitivity estimate of 22% (95% CI: 0.11–0.37). Of the 10 samples that tested positive for metabarcoding, eight of them tested positive at all three replicates, with the remaining two samples testing positive at one or two replicates each. The 10 positive-testing samples included all five of the samples testing positive for qPCR in addition to five that did not test positive in the qPCR assays. The sensitivity estimate for metabarcoding was twice as high as that estimated for qPCR, representing a difference of 11.1% on average, which was marginally significant (McNemar’s test *p* = 0.063).

Sensitivity was much higher when considering only the 11 foxes confirmed by skin scraping to have active cases of mange, with nine of the positive tests corresponding to one of these samples (Table S2). For this subset, the sensitivity was estimated at 81.8% (95% CI: 0.48–0.98), which was 36.4% higher on average than the corresponding estimate for qPCR, although the difference was not statistically significant (McNemar’s test *p* = 0.13). Only one of the 34 putative cases tested positive.

Based on the conservative estimate utilizing all 45 putative or confirmed mange-infested samples, the true prevalence values are expected to be nearly 5-times higher than what would be estimated based on the metabarcoding of scats. These relationships imply a detection probability in a population with 10% prevalence of 97% for 100 scats or >99.9% for 200 scats.

## 4. Discussion

To assess the feasibility of a noninvasive fecal DNA approach to detecting mange in kit fox populations, we tested qPCR and metabarcoding methods to detect sarcoptic mite DNA in San Joaquin kit fox scats. Each approach was validated on serial dilutions of sarcoptic mite DNA and scat samples collected from San Joaquin kit foxes identified as either infested or not, including a subset confirmed to have active infestations at the time of scat sampling. To augment negative samples and increase precision of specificity estimates, we included scat samples from kit foxes located in Ciervo–Panoche population, all of which tested negative in all tests from both approaches. Both assays exhibited perfect specificities, agreeing with previous findings using the same primers [11].

While our estimated sensitivities using all putative and confirmed mange cases were not as high as anticipated [9,10], they could have been biased by overrepresentation of foxes with low mite burdens. Some of the visually diagnosed cases could also have been misdiagnosed as well, but the advanced stages that did not receive skin scrapings (*n* = 9) were treated for mange and their clinical signs were resolved, supporting the validity of at least some of the visual diagnoses. Although we did not quantify mite burden explicitly, many (*n* = 26) of the putative mange cases diagnosed visually reflected early-stage infestations (Class I) likely to correspond to low mite burdens (although one visually diagnosed Class I case also tested positive). Most of these foxes were sampled during a decline phase of the epizootic when teams were better able to catch and treat cases early than during the increase or peak phases. Positive samples in our study tended to test positive across replicates for both qPCR and metabarcoding assays, which indicates that the tests were highly repeatable, further supporting that the lack of detection in samples from mange-infested foxes was due to a lack of mite DNA in the sample (i.e., concentration below the detection limit) rather than poor performance of the tests themselves. Indeed, our estimates based solely on scats from foxes with skin scrapings confirming active infestations were much higher, albeit with large confidence intervals due to the small sample size; the qPCR sensitivity estimate (0.46, 95% CI: 0.17–0.77) was similar to those of other fecal-DNA-based studies of mite detection in felids and canids [9,10], and that for the metabarcoding assay was even higher (0.82, 95% CI: 0.48–0.98).

Although it is also possible that more technical variables affected sensitivity, we suspect these played a relatively insignificant role compared to mite burden or at least mite DNA in scats. The storage time scats spent in the freezer before analysis in our study was up to 12 years, but we found no evidence that this affected results, as the only two skin-scraping-confirmed mange cases that did not test positive with metabarcoding were among the oldest and most recently collected (2014, 2022), whereas the nine positive cases also ranged over those same years (Table S2). Although the digestion process causes DNA from ingesta to be mixed to some extent throughout a scat, this distribution may not be entirely homogeneous, in which case the use of a subsample of scats could also contribute to false negative results. More generally, DNA quality/quantity and occurrence of PCR inhibitors can vary in fecal DNA extracts, but these tend to be stochastic and are therefore incorporated in the sensitivity estimates (i.e., not expected to bias tests).

Other biological factors besides mite burden could include phase of the infestation, or the behavioral tendencies of foxes to self-groom and ingest mites. For example, detection of notoedric mites in fecal DNA of bobcats (*Lynx rufus*) using a different marker [9] achieved a higher sensitivity (about 2× higher) than our conservative estimate or that of a wolf study [10] with respect to sarcoptic mange using the same marker as used in our study. This difference could be due to the comparably more intense grooming behaviors and higher parasitic load in feces of felids than canids. Depending on grooming habits and other behavioral factors, our test could potentially be useful in other wildlife for which sarcoptic mange is a problem, such as black bears (*Ursus americanus*) [20].

As a secondary objective, we also sought to determine whether metabarcoding could be as or more sensitive as/than qPCR. Although the difference between sensitivity estimates between qPCR and metabarcoding methods was marginally significant when assessed as a binary test, the superior performance of the metabarcoding method was additionally supported by the lower levels of detection (DNA concentration) of metabarcoding compared to qPCR as well as the observation that both estimates fell outside the 95% confidence interval of the other. The higher sensitivity of metabarcoding than qPCR was not necessarily anticipated as similar comparisons between the ability of these methods to detect trace levels of DNA have met with variable results. In one study attempting to detect DNA from a fish parasite in lake water samples, the two methods performed similarly [21]. In another study attempting to detect DNA of an amphibian in pond-water samples, qPCR proved to be slightly more sensitive than metabarcoding [22]. In addition to the better performance of metabarcoding than qPCR in our study, several other advantages of metabarcoding in general recommend it, including easier start-up and optimization and ability to simultaneously assay for other components of the scat, including other pathogens, diet, and microbiome [18,21,23].

The intended purpose of the assays we investigated was to detect the occurrence of mites in a fox population by testing multiple samples rather than as a test for individual cases, which requires a much higher sensitivity to be useful. Our estimates of sensitivity and specificity allow for designs of surveys in terms of the numbers of scats required to achieve a given probability of detection in the population assuming some minimum prevalence (e.g., 10%). This calculation may also be conservative if, as we discuss above, our sensitivity was underestimated, but it should be tested empirically and refined accordingly regardless.

While we did not expect our fecal assays to have sufficiently high sensitivities to be useful as a reliable test for mange in individuals, our metabarcoding results on the 11 skin-scraping-confirmed mange-infested foxes suggest the possibility that it could be. Clearly, however, the sample size was too small to be conclusive and additional research is needed to obtain more precise estimates based on similar confirmed cases. Additionally, future studies should quantify mite burden and antibody levels as correlates.

## 5. Conclusions

Sarcoptic mange continues to impact and threaten the persistence of the endangered San Joaquin kit fox population in Bakersfield, California, USA. The metabarcoding assay piloted here can serve as an additional tool to identify the presence of mange in populations, especially in non-urban and at-risk kit fox populations where mange could spread but where resources preclude intensive camera and live-trap monitoring. Our tests exhibited low sensitivities when estimated from suspected infestations unconfirmed as active, but much higher sensitivities in the subsample of individuals confirmed by skin scrapings to have active infestations, suggesting that it may be most useful in detecting active mange cases, particularly those with higher mite burdens. If so, populations in the increase or peak phases of outbreaks that have not been affected by treatment interventions may be most benefited by these tools. The primary intended purpose of this method was as a means of detecting the presence of mange within populations, which can be achieved by testing sufficient numbers of scats even assuming our conservatively estimated sensitivity estimate is based on all known or suspected mange cases. Although we only tested the assays on kit foxes, they would likely be usable for other mammals that are known to be susceptible to sarcoptic mange and which could serve as reservoirs for the mite, such as coyotes (*Canis latrans*) or nonnative red foxes (*V. vulpes*), and, pending evaluation, potentially non-canids (e.g., black bears) as well.

## Data Availability

The original contributions presented in this study are included in the article/Supplementary Materials. Further inquiries can be directed to the corresponding author.

## Supplementary Materials

The following supporting information can be downloaded at: https://www.mdpi.com/article/10.3390/ani16172740/s1, Table S1: Scat samples from 45 mange-infested and 44 uninfested kit foxes used to evaluate qPCR and metabarcoding tests; Table S2: Results of metabarcoding and qPCR analysis of scat samples from 45 mange-infested and 44 uninfested kit foxes run in triplicate1, showing the resulting No. of sequencing reads for each replicate and the test result based on them; Table S3: Results of metabarcoding analysis of 20 negative controls and 16 positive controls at a range of sarcoptic mite (*Sarcoptes scabiei*) DNA concentrations, showing the resulting No. of sequencing reads.
